# Effekte einer Tilgungsstreckung für coronabedingte Staatsschulden

**DOI:** 10.1007/s10273-022-3137-0

**Published:** 2022-03-19

**Authors:** Michael Broer

**Affiliations:** Fakultät Wirtschaft — Campus Wolfsburg, Ostfalia Hochschule für angewandte Wissenschaften, Siegfried-Ehlers-Str. 1, 38440 Wolfsburg, Deutschland

**Keywords:** H60, H62, H63

## Abstract

Der Koalitionsvertrag erschwert Steuererhöhungen. Die Bundesregierung benötigt aber Einnahmen für politisch erwünschte Projekte. Aus diesem Grund plant die Regierung, den Tilgungsplan für die Coronakredite zu strecken. Diese Politik mindert die Tilgungsbelastung in der Gegenwart und schafft somit finanzielle Spielräume für Ausgaben. Steigen zukünftig die Renditen der Staatsanleihen, so erhöht sich aufgrund der Verlängerung der Tilgungsdauer die Zinsbelastung. Werden die durch die Tilgungsstreckung gewonnenen finanziellen Spielräume für Investitionen verwendet, so erben zukünftige Generationen Schulden und Vermögenswerte. Tatsächlich liegen die investiven Ausgaben des Bundes in den vergangenen zwei Jahren bei 11,3%. Würde dies auch für diese Mittel gelten, käme es zu einer starken Belastung der zukünftigen Generation und einem Verstoß gegen die Generationengerechtigkeit.

## Abstract (en)

The coalition agreement prevents tax increases. However, the federal government needs income for politically desirable projects. For this reason, the government is planning to stretch the re-payment plan for the corona loans. This policy reduces the repayment burden in the present thereby creating financial leeway for spending. If the yields on government bonds rise in the future, the interest burden will increase due to the extension of the repayment period. If the financial leeway is used for investments, future generations will inherit debts and assets. In fact, federal investment spending over the past two years has been 11.3 %. If this rate is also applied to these funds, there would be a heavy burden on the future generation. This would be a violation of inter-temporal equivalence.

Die finanziellen Spielräume der neuen Bundesregierung sind aufgrund des Andauerns der COVID-19-Pandemie und der damit verbundenen Verschlechterung der zukünftigen wirtschaftlichen Entwicklung noch immer sehr begrenzt (Ifo, 2021). Dies ist insofern für die Koalitionär:innen problematisch, als eine Erhöhung der Steuereinnahmen zumindest bei den wichtigen und aufkommensstarken Steuern laut Ausführungen des Sondierungspapiers nicht möglich ist. Dort heißt es: „Wir werden keine neuen Substanzsteuern einführen und Steuern wie zum Beispiel die Einkommen-, Unternehmens- oder Mehrwertsteuer nicht erhöhen“ (Sondierungspapier, 2021, 10).

Um sich trotzdem finanzielle Spielräume für „Zukunftsinvestitionen“ zu verschaffen, greift die Regierung auf vorhandene Kreditermächtigungen zurück (Bundesministerium der Finanzen, 2021b). Mittels eines zweiten Nachtragshaushalts für das Jahr 2021 soll die Kreditaufnahme um weitere 60 Mrd. Euro erhöht werden (Deutscher Bundestag, 2021a, 6). Die Regierung greift dabei auf Art. 115 Abs. 2 Satz 6 GG zurück. Ob diese Vorgehensweise verfassungskonform ist, ist umstritten. Dieser Aspekt des Art. 115 GG soll hier nicht geprüft werden, dies wird vermutlich das Bundesverfassungsgericht demnächst tun (Tagesschau, 2021a).

* Der Verfasser dankt K.-D. Henke und H. Zimmermann für hilfreiche Anmerkungen und Kommentare.

**Prof. Dr. Michael Broer** ist Professor für Volkswirtschaftslehre an der Ostfalia Hochschule für angewandte Wissenschaften in Wolfsburg.

## Gravierende Änderungen der Tilgungen

Nicht thematisiert wurde bisher, dass auch die Tilgung als Instrument eingesetzt werden kann, um politisch erwünschte Ausgabenspielräume zu schaffen. Nachfolgend sollen Art 115 Abs. 2 Satz 7 und 8 GG und die damit verbundenen Effekte analysiert werden, die sich bei der Tilgung der im Zusammenhang mit der COVID-19-Pandemie aufgenommenen Kredite ergeben; denn auch die Tilgung der Staatsschulden war in der Vergangenheit lange Zeit ein wichtiges Thema (Andel, 1964). Die Tilgung wird auch in den kommenden Jahrzehnten bedeutsam sein, zumal laut Koalitionsvertrag in diesem Bereich Änderungen vorgenommen werden sollen, die bisher aber noch nicht in der Öffentlichkeit diskutiert wurden. Es geht um die Frage des Zeithorizonts bei der Tilgung von Staatsschulden. Art 115 Abs. 2 Satz 7 und 8 GG fordert allein, dass ein Tilgungsplan aufgestellt wird und die Tilgung in einem angemessenen Zeitraum zu erfolgen hat. Auch die Gesetzesbegründung gibt hier keine weiteren Hinweise, dort heißt es einfach: „Welcher Zeitraum als angemessen für die Rückführung anzusehen ist, hat das Parlament in Ansehung der Größenordnung der erhöhten Kreditaufnahme sowie der konkreten konjunkturellen Situation zu entscheiden“ (Deutscher Bundestag, 2009, 13).

Die Entscheidung über den Tilgungszeitraum allein und vollständig in das Ermessen der Politiker:innen zu stellen, ist vor dem Hintergrund der neuen ökonomischen Theorie der Politik und der eigennutzmaximierenden Politiker:innen problematisch (Downs, 1968). Es ist zu erwarten, dass im Falle eines (verfassungsrechtlichen) Tilgungszwangs die Politiker:innen den Tilgungszeitraum maximieren werden, damit in der Gegenwart der finanzielle Ausgabespielraum für wählerstimmenmaximierende Ausgaben möglichst wenig durch jährliche Tilgungsraten beeinträchtigt wird. Lasten werden also in die Zukunft verlagert. Ein solches Verhalten wurde im Zusammenhang mit dem Klimaschutzgesetz des Bundes für verfassungswidrig erklärt (Bundesverfassungsgericht, 2021).

Der im Zusammenhang mit der Änderung der Tilgungszahlungen sehr wichtige Hinweis ist im Koalitionsvertrag etwas versteckt. Bezüglich der Haushaltspolitik steht der folgende Satz: „Die Tilgung passen wir an die Tilgungsfristen der EU-Coronahilfen aus dem Programm Next Generation EU an“ (Koalitionsvertrag, 2021, 160). Was auf den ersten Blick als eine an der EU ausgerichtete Politik erscheinen mag, ist tatsächlich eine zeitliche Streckung der Tilgungszahlungen in Deutschland. Wird die Tilgung gestreckt, so führt dies in den ersten Jahren zu einer geringeren Belastung und in den späteren Jahren zu einer höheren Belastung als ursprünglich geplant. Es kommt somit zu einer Verlagerung der Lasten in die Zukunft und einer Ausweitung des Ausgabespielraums in der Gegenwart. Dieser Sachverhalt soll nunmehr genauer analysiert werden.

## Derzeitiger Tilgungsplan

Nach den vom Bundesministerium der Finanzen im September veröffentlichten Werten würde die Tilgungsleistungen für alle im Rahmen der COVID-19-Pandemie aufgenommenen Kredite im Jahr 2042 abgeschlossen sein. Dass es sich bei diesen Angaben über die jährlichen Tilgungsleistungen nur für die Kredite des Jahres 2020 um Ist-Daten handelt, nicht aber für die Jahre 2021 und 2022, spielt keine Rolle, da allein die ungefähre Größenordnung ausreicht, um die auftretenden Effekte abschätzen zu können. Zu beachten ist allerdings, dass nicht zwangsläufig der jeweilige Betrag netto getilgt werden muss, die Werte können auch als eine Verringerung des ansonsten möglichen Verschuldungsrahmens interpretiert werden (Bundesministerium der Finanzen, 2021c, 40). Nachfolgend wird aber der Begriff Tilgung verwendet.

Die Tilgung für die im Jahr 2020 aufgenommenen Kredite in Höhe von rund 42 Mrd. Euro soll im Jahr 2023 beginnen und nach 20 Jahren abgeschlossen sein (Deutscher Bundestag, 2020). Der jährliche Tilgungsbetrag liegt bei 2,1 Mrd. Euro. Bei den in den Jahren 2021 aufgenommenen Krediten sind laut Gesetz der Tilgungsbeginn und die Tilgungszeit so ausgestaltet, dass auch diese im Jahr 2042 getilgt sein werden (Deutscher Bundestag, 2021b, 1). Es würden sich nach der derzeitigen Planung jährliche Tilgungsraten in der Zeit von 2026 bis 2042 von 12,7 Mrd. Euro (Kredite aus 2021) bzw. 5,8 Mrd. Euro (Kredite aus 2022) ergeben (Bundesministerium der Finanzen, 2021c, 41). Letztlich würde die jährliche Tilgungsbelastung zwischen 2023 und 2042 wie folgt aussehen:

- Jährliche Tilgung in den Jahren von 2023 bis 2025: 2,1 Mrd. Euro.
- Jährliche Tilgung in den Jahren von 2026 und 2042: 20,6 Mrd. Euro.

Dieser Plan wäre für die derzeitige Koalition mit einer jährlichen Belastung von 2,1 Mrd. Euro verbunden. Sollte, wie Kanzler Olaf Scholz bekundet hat, diese Koalition nicht nur in der 20. Legislaturperiode regieren (Tagesschau, 2021b), so wäre der Ausgabenspielraum in der 21. Legislaturperiode durch die jährlichen Tilgungszahlungen von fast 21 Mrd. Euro deutlich eingeschränkt. Zu beachten ist, dass in dieser Darstellung allein die Tilgungen, nicht die Zinszahlungen berücksichtigt sind. Derzeit erhält der Bund Zinsen für die Kredite. In der Darstellung der Regierung werden diese Zinseinnahmen allerdings nicht für die Tilgung verwendet, was zu einer schnelleren Tilgung der Gesamtschuld führen würde. Es ist insofern davon auszugehen, dass diese Einnahmen dem allgemeinen Haushalt zufließen.

## Drei Änderungsszenarien für den Tilgungsplan und deren Effekte auf die Tilgungen

Nach dem aktuellen Plan würde die Regierung jährlich 2,1 Mrd. Euro und ab 2026 (bis einschließlich 2042) 20,6 Mrd. Euro tilgen. Unklar ist, wie genau die von der Koalition vorgesehene Anpassung der Tilgungszahlungen an das Programm „Next Generation EU“ erfolgen soll. Klar ist, dass Änderungen mit einfacher Mehrheit im Bundestag beschlossen werden können (Feld et al., 2021, 338). Mehrere Szenarien sind für die Änderungen der Tilgungen denkbar. Nachfolgend werden zunächst allein die Wirkungen dieser Szenarien auf die jährliche Tilgungszahlung betrachtet; der Zusammenhang zwischen der Höhe der Zinszahlungen und der Laufzeit der Kredite ebenso wie die Effekte der Inflation werden hingegen aus Vereinfachungsgründen ausgeblendet.[1]

1 Zum Zusammenhang von Kreditlaufzeit und Zinshöhe, Größen, die im Rahmen des Debt Managements beeinflusst werden können, siehe Wissenschaftlicher Beirat beim Bundesministerium der Finanzen (2021).

Eine Änderung des Tilgungsplans besteht darin, sich vollumfänglich an der EU zu orientieren. In diesem Fall würden die Kredite nicht zwischen 2023 und 2042 getilgt, sondern wie es für die von der EU aufgenommenen Kredite vorgesehen ist zwischen 2028 und 2058 (Szenario 1) (Europäische Union, 2021). Die Tilgungsraten würden in diesem Fall jährlich bei 11,5 Mrd. Euro liegen. Alternativ könnte allein die von der EU vorgesehene Tilgungsdauer übernommen werden. In diesem Fall würden alle Kredite einheitlich über 31 Jahre getilgt werden; der Tilgungsbeginn wäre weiterhin 2023, das Ende 2053 (Szenario 2). In diesem Fall würde im Jahr 2023 mit einer Tilgung von 1,4 Mrd. Euro begonnen werden, in der Zeit von 2026 bis 2053 wären es 11,5 Mrd. Euro jährlich. In den letzten drei Tilgungsjahren (2054 bis 2056) wäre der jährliche Tilgungsbetrag 10,1 Mrd. Euro. Die letzte hier betrachtete Möglichkeit bestünde darin, allein das Enddatum der Tilgung von der EU zu übernehmen. Auch hier wäre der Tilgungsbeginn 2023, das Ende aber erst 2058, sodass die Tilgungsdauer der 2020 aufgenommenen Kredite 36 Jahre betragen würde (Szenario 3). Statt nach der bisherigen Planung maximal über 20 Jahre oder gar 17 Jahre die Tilgungsleistungen zu erbringen, würde der Zeitraum 36 (für die 42 Mrd. Euro aus 2021) bzw. Jahre 33 (für die Kredite aus 2021 und 2022) betragen. Im Vergleich zu dem von den Bundesländern geplanten Zeitraum wäre diese Tilgungsdauer von bis zu 36 Jahren überdurchschnittlich lang (Nitschke, 2021, 4).

## Erhöhung der Ausgabespielräume durch die Tilgungsszenarien

Abbildung 1 zeigt die finanziellen Vorteile der drei hier dargestellten denkbaren Änderungen gegenüber der ursprünglichen Planung auf, welche aus den Formulierungen des Koalitionsvertrags ableitbar sind. Würde sich die Bundesregierung sowohl bei der Tilgungsdauer als auch beim Startpunkt der Tilgung an die EU-Vorgehensweise orientieren (Szenario 1), so würde statt im Jahr 2023 erst im Jahr 2028 mit der Tilgung begonnen. Die Tilgung würde erst 2058 und nicht bereits 2042 enden bei einem jährlichen Tilgungsbetrag in Höhe von 11,5 Mrd. Euro. Die Folge dieser Maßnahme wäre, dass sich in den Jahren 2023 bis 2027 der finanzielle jährliche Spielraum durch die unterlassene Tilgung erhöhen würde. In den ersten drei Jahren betrüge der gewonnene Spielraum jährlich 2,1 Mrd. Euro und in den Jahren 2026 und 2027 jeweils 20,6 Mrd. Euro. In der Zeit von 2028 (neuer Tilgungsbeginn) bis 2042 würde sich jährlich ein finanzieller Vorteil in Höhe von 9,1 Mrd. Euro ergeben. In den restlichen Jahren bis 2058 hingegen käme es zu einer Einschränkung des Ausgabespielraums um jährlich 11,5 Mrd. Euro. Wird allein der Tilgungszeitraum auf 31 Jahre erhöht, wie beim EU-Programm vorgesehen, würde in den Jahren 2023 bis 2025 die jährliche finanzielle Entlastung gegenüber der

Abbildung 1

**Änderung der Ausgabespielräume gegenüber geltendem Recht**

in Mrd. Euro

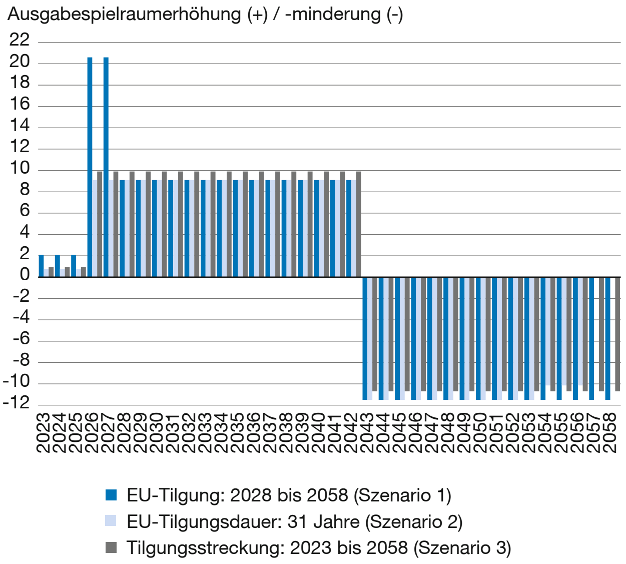

Quelle: eigene Darstellung.

Planung bei 0,7 Mrd. Euro liegen (Szenario 2). Zwischen 2026 und 2042 wären es 9,1 Mrd. Euro. Eine Mehrbelastung würde ab 2043 auftreten und zunächst bei 11,5 Mrd. Euro liegen; für die letzten drei Tilgungsjahre (2054 und 2056) wären es 10,1 Mrd. Euro. Das Szenario 3 besteht darin, die Tilgungen der durch die COVID-19-Pandemie in den Jahren 2020, 2021 und 2022 initiierten Kredite bis 2058 zu strecken. Wie schon dargelegt, würde die Tilgung in den Jahren 2023 bis zum Jahr 2025 auf 1,2 Mrd. Euro sinken. Der finanzielle Spielraum, der sich durch diese Maßnahme ergeben würde, würde bei jährlich 0,9 Mrd. Euro liegen und für die drei Jahre bis 2025 ein Volumen von 2,7 Mrd. Euro ausmachen. In den folgenden Jahren bis einschließlich 2042 ergäbe sich ein finanzieller Vorteil von jährlich 9,9 Mrd. Euro. Dafür müssten in den Jahren 2043 bis 2058 jährlich 10,7 Mrd. Euro zusätzlich gegenüber dem ursprünglichen Plan getilgt werden.

Aus Abbildung 1 ergibt sich für die Bundesregierung in der aktuellen Legislaturperiode eine Erhöhung des Ausgabespieltraums in Höhe von maximal 6,3 Mrd. Euro (Szenario 1). Wird noch die 21. Legislaturperiode hinzugerechnet, kämen noch weitere 59,4 Mrd. Euro hinzu. Sollte hingegen allein der Tilgungszeitraum an die EU-Tilgungsdauer angepasst werden (31 Jahre), so könnten in der 20. Legislaturperiode 2,1 Mrd. Euro zusätzlich ausgegeben werden und in der 21. Legislaturperiode 39,4 Mrd. Euro (Szenario 2). Die Werte für Szenario 3 liegen zwischen diesen Angaben. Für die nachfolgenden Betrachtungen wird allein das Szenario 1 (EU-Tilgung) betrachtet, da sich in diesem Fall für die Regierung der größte finanzielle Vorteil für die Gegenwart ergibt.

## Debt Management

Die Höhe der Zinszahlung für ein Kreditvolumen ist ceteris paribus von der Laufzeit des Kredits abhängig. Dabei ist festzustellen, dass mit der Verkürzung der Laufzeit auch der zu zahlende Zinssatz sinkt bzw. in der aktuellen Situation mit negativen Zinssätzen die Einnahmen steigen. So betrug Ende 2020 / Anfang 2021 z. B. die Renditedifferenz zwischen einer zweijährigen Laufzeit bei deutschen Staatsanleihen und einer zehnjährigen Laufzeit 0,2 Prozentpunkte. Dieser Wert lag am Ende des Jahres 2021 bei 0,4 Prozentpunkten und ist damit immer noch sehr gering; Ende 2009 / Anfang 2021 gab es Zinsdifferenzen von über 2,4 Prozentpunkten (o. V., 2021a). Für den Bund ergab sich bei Krediten mit einer zehnjährigen Laufzeit ein Zins von -0,15 % (o. V., 2021b); bei zweijähriger Laufzeit ergab sich aufgrund der Zinsdifferenz von -0,4 Prozentpunkten ein Zins von -0,65 %. Die Bundesregierung erzielt somit beim derzeitigen Zins Einnahmen aus der Staatsverschuldung. Durch diese von der Laufzeit der Kredite abhängige Zinsdifferenz ergeben sich für den Staat Möglichkeiten die Zinszahlungen unter anderem durch Veränderung der Laufzeiten der Kredite zu beeinflussen. Diese als Debt Management (Zimmermann et al., 2021, 150 f.) bezeichnete Politik kann bei der aktuell enormen Kreditaufnahme zu deutlichen Veränderungen bei der Haushaltsbelastung durch Zinsausgaben führen.

Deutschland hat sich im Zusammenhang mit der Coronakrise vor allem kurzfristig verschuldet. Während im Jahr 2019 noch 52 % der emittierten Wertpapiere eine Laufzeit von mehr als zwei Jahren hatten, sind es im Jahr 2020 nur 41,7 % und 2021 37,4 %. Für das Jahr 2022 ergibt sich nach derzeitigen Plänen ein Anteil von 32,9 %. Im Gegenzug hat sich der Anteil der Wertpapiere mit einer Laufzeit von bis zu einem Jahr von 21,3 % auf 50,7 % erhöht; in absoluten Werten sind es statt 43 Mrd. Euro (2019) nun 238 Mrd. Euro (2021). Werden zudem noch die Wertpapiere mit einer Laufzeit von zwei Jahren einbezogen, so ergibt sich ein prognostizierter Anstieg für kurzfristige Kredite mit einer Laufzeit von maximal zwei Jahren auf 67,0 % im Jahr 2022 (vgl. Abbildung 2).

Abbildung 2
**Emissionsvolumen von Bundeswertpapieren**
Anteil in %

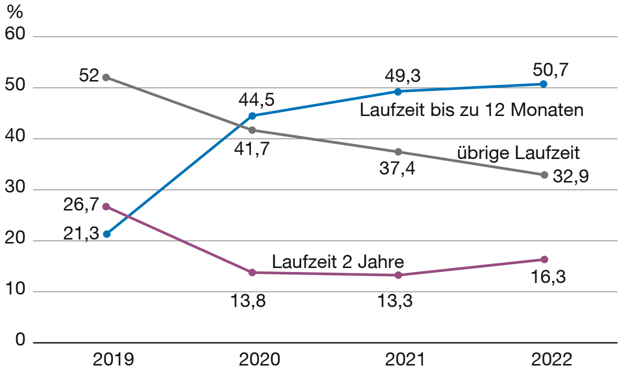

Quelle: https://www.deutsche-finanzagentur.de/de/institutionelle-investoren/primaermarkt/ (21. Januar 2022).

## Zinsänderungsrisiko

Dem aktuellen Zinsvorteil einer kurzfristigen Verschuldung ist allerdings das Zinsänderungsrisiko gegenüber zu stellen. Steigen etwa die Renditen für Staatsanleihen, so verteuert sich die Refinanzierung bei kurzfristigen Krediten sehr schnell, während es bei Wertpapieren mit einer zehnjährigen Laufzeit zunächst Planungssicherheit für einen längeren Zeitraum gibt. Da die Zinsdifferenz derzeit im historischen Vergleich als gering gelten kann, verliert dieser Zinsvorteil vor dem Hintergrund des Zinsänderungsrisikos an Gewicht, zumal das Zinsänderungsrisiko derzeit in den USA (Rottwilm, 2022) und auch in Deutschland (Frühauf, 2022) aktuell ist. Vor dem Hintergrund aktuell steigender Renditen ist das Zinsänderungsrisiko virulent. Der Bund sollte also von einer kurz- zu einer längerfristigen Verschuldung wechseln (Wissenschaftlicher Beirat, 2021, 17 f.).

Nach dem ursprünglichen Tilgungsplan der Bundesregierung wäre die Tilgung der 356,5 Mrd. Euro bis zum Jahr 2042 abgeschlossen. Nach dem hier unterstellten Szenario, welches zeitlich dem Tilgungsplan der EU entspricht, wären zu diesem Zeitpunkt erst 172,5 Mrd. Euro getilgt. Für die Jahre 2043 bis 2058 wären noch Zahlungen in Höhe von 184 Mrd. Euro zu erbringen (vgl. Abbildung 3, linke Seite). Würde das derzeitige Renditeniveau von -0,65 % erhalten bleiben bis zum Jahr 2058, so würden die Politiker:innen statt Einnahmen in Höhe von 27,4 Mrd. Euro solche in Höhe von 48,7 Mrd. Euro erzielen, also zusätzliche Einnahmen in Höhe von 21,3 Mrd. Euro (vgl. Abbildung 3, Mitte). Würde hingegen die Regierung positive Renditen zahlen müssen, so würde es schnell zu hohen zusätzlichen Belastungen kommen. Bei einer Rendite von 2,5 % für die Staatsanleihen, ein Wert der in der Vergangenheit nicht ungewöhnlich gewesen ist, würde die gesamte Zinsbelastung durch die Änderung der Tilgungsraten von 105,4 Mrd. Euro (Tilgung 2023 bis 2042) auf 187,2 Mrd. Euro (Tilgung 2028 bis 2058) steigen, wovon 39,1 Mrd. Euro auf die Jahre 2043 bis 2058 entfallen. In der Zeit von 2023 bis 2042 würde es zu einem Belastungsanstieg um 42,7 Mrd. Euro kommen (vgl. Abbildung 3, rechte Seite).

Abbildung 3
**Status quo und mögliche Änderung durch die Ampel: Zins- und Tilgungsleistungen 2023 bis 2058**
Belastung (+) / Entlastung (-) in Mrd. Euro

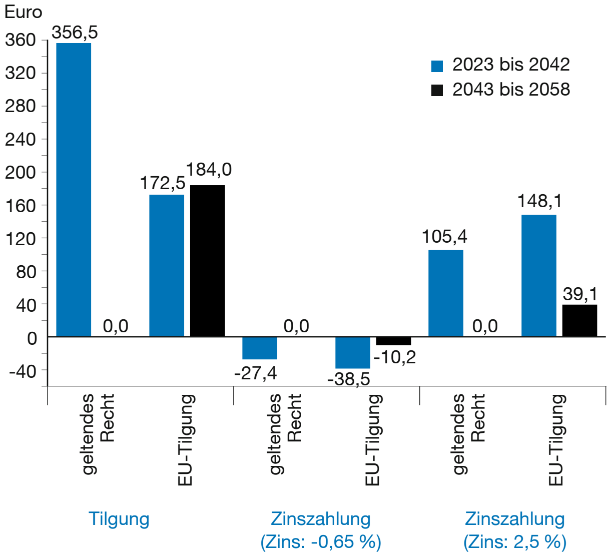

Quelle: eigene Darstellung.

## Verwendung der durch Tilgungsstreckung gewonnenen Mittel

Bisher ging es vor allem um den Effekt der Tilgungsplanänderung auf die Zinszahlungen. Nun soll eine Generationenbetrachtung vorgenommen werden. Wird der ursprüngliche Tilgungsplan als generationengerecht angesehen, so hätte die Generation, die durch Kurzarbeitergeld und diverse Überbrückungshilfen von den Coronamaßnahmen der Bundesregierung profitiert, diese 356,5 Mrd. Euro auch über Zins- und Tilgungszahlungen bis zum Jahr 2042 finanziert, anders im Fall der Übernahme des EU-Tilgungszeitraums.

Wird der gewonnene Einnahmenspielraum vollumfänglich für konsumtive oder investive Ausgaben verwendet, so dürfte die Beurteilung einfach sein. Im ersten Fall würden ab 2043 allein Tilgungsleistungen zu erbringen sein, denen keinerlei Vermögenswerte gegenüberstehen. Zwar würden bei unveränderten Zinssatz von -0,65 % den Menschen auch weiterhin Einnahmen aus den dann noch zu tilgenden coronabedingten Schulden zufließen, doch diese 10,2 Mrd. Euro stehen in keinem Verhältnis zu den zu erbringenden Tilgungsleistungen in Höhe von 184 Mrd. Euro. Werden in den Jahren 2023 bis 2042 die 184 Mrd. Euro vollumfänglich für Investitionen verwendet, so stünden diese ab 2043 für die zukünftige Generation zur Verfügung und im Gegenzug wären im gleichen Umfang Tilgungsleistungen zu erbringen.[2] Diese Vorgehensweise entspricht dem Gedanken der „Goldenen Regel“ in Art. 115 Abs. 1 Satz 2 GG (alte Fassung), wodurch intertemporale Gerechtigkeit bzw. Äquivalenz vom Grundsatz her erreichbar ist (Sachverständigenrat, 2007, 49-54). Hinzu kommen beim Zinssatz von -0,65 % die bereits erwähnten Einnahmen in Höhe von 10,2 Mrd. Euro. Die Renditen öffentlicher Investitionen sind schwierig zu ermitteln. Sie können positiv (Krebs und Scheffel, 2017) sein, sie können aber auch wie z. B. bei Flughäfen (Kassel-Calden oder den BER in Berlin) und anderen staatlichen Investitionen negativ sein. Auch bei den Mitteln, die den Strukturwandel in der Lausitz unterstützen sollen, gibt es Zweifel daran, ob die Mittel zukunftsweisend verwendet werden (Ragnitz, 2021). Hier wird unterstellt, dass die Renditen in Form von zusätzlichen Steuereinnahmen ausreichen würden, um die Zinszahlungen, etwa bei einer Rendite der Staatsanleihen von 2,5 %, zu erbringen. Zu einer Lastverschiebung würde es somit nicht kommen, wenn die gesamten 184 Mrd. Euro investiert werden.

## Nur anteilige investive Verwendung des gewonnenen Ausgabespielraums

Die bisherige Darstellung ging davon aus, dass der durch die Tilgungsstreckung gewonnene Einnahmenspielraum vollständig für konsumtive oder investive Ausgaben verwendet wird, wobei es im ersten Fall eine Lastverschiebung stattfinden würde, nicht aber im letztgenannten Fall. Beide Fälle sind unwahrscheinlich. Eher ist damit zu rechnen, dass die Regierung nur einen (kleinen) Teil der Mittel für investive Ausgaben verwenden wird.

Dass Regierungen trotz anderslautender Aussagen vor allem die konsumtiven Ausgaben priorisieren, kann aus der Vergangenheit und auch dem Koalitionsvertrag der neuen Bundesregierung entnommen werden. So hat die Koalition aus CDU/CSU und SPD sowohl die Mütterrente als auch die Rente mit 63 eingeführt, obwohl Fachleute davon abrieten, da beide Maßnahmen zu einer Belastung der zukünftigen Generationen führen (Sachverständigenrat, 2014, 559-571). Im aktuellen Koalitionsvertrag ist festgehalten, dass die Steuerzuschüsse für die Gesetzliche Kranken- und die Gesetzliche Pflegeversicherung erhöht werden. Zudem soll das Rentenniveau nicht sinken und das Renteneintrittsalter nicht steigen, was nur durch Steuerzuschüsse oder Ausgabenkürzungen an anderer Stelle erreichbar sein dürfte (Koalitionsvertrag, 2021, 6, 66, 73, 80 und 87). Gerade von den letztgenannten Maßnahmen profitieren wiederum vor allem die Rentner:innen und die rentennahen Jahrgänge. Vor diesem Hintergrund wird nachfolgend unterstellt, dass

2 Der Vermögensverzehr in Form von Abschreibungen wird in der auf Bundesebene vorherrschenden Kameralistik nicht richtig erfasst.

sich die Regierung bei der investiven Verwendung der finanziellen Mittel, die durch die Streckung der Tilgung gewonnen werden können, an den Investitionsquoten der letzten Haushaltsjahre orientieren wird.

Die Sachinvestitionen des Bundes machten in den Jahren 2020 und 2021 einen Anteil an den Gesamtausgaben in Höhe von maximal 3,3 % aus. Werden noch die Investitionszuschüsse, die vor allem an die Bundesländer fließen, einbezogen, so ergibt sich für die investiven Ausgaben ein Anteil von rund 11,3 % (Bundesministerium der Finanzen, 2021a, 216). Wird die Quote der investiven Ausgaben auch für die 184 Mrd. Euro unterstellt, so würden tatsächlich nur rund 20,8 Mrd. Euro für Investitionen verwendet. Werden von den 184 Mrd. Euro nur 20,8 Mrd. Euro für investive Ausgaben aufgewendet, so würden in den Jahren 2043 bis 2058 der zukünftigen Generation Schulden in Höhe der Differenz der beiden gerade genannten Werte „vererbt". Von diesem Wert in Höhe von 163,2 Mrd. Euro müssten noch die Zinserträge in Höhe von 10,2 Mrd. Euro abgezogen werden, die derzeit beim Zins von -0,65 % erzielt werden, sodass die Last letztlich 153 Mrd. Euro betragen würde.[3] Bei einem Zinssatz von 2,5 % für die aufgenommenen Kredite müssten hingegen zusätzlich zu den 163,2 Mrd. Euro noch die Mehrausgaben für Zinsen als Folge der Tilgungsstreckung in Höhe von 81,8 Mrd. Euro (187,2 Mrd. Euro – 105,4 Mrd. Euro) (vgl. Abbildung 3, rechte Hälfte) erbracht werden. Vereinfachend wird nun unterstellt, dass die für die Investitionen in Höhe von 20,8 Mrd. Euro benötigten Mittel aus den eingesparten Tilgungsleistungen des Jahres 2025 (0,2 Mrd. Euro) und 2026 (20,6 Mrd. Euro) erbracht werden. Bei einem Zinssatz von -0,65 % müssten die öffentlichen Investitionen in Höhe von 20,8 Mrd. Euro in den 17 Jahren bis Ende 2042 jährliche Renditen in Höhe rund 13 % erbringen, damit es nicht zu einer Belastungsverschiebung kommt und sich die benötigten 153 Mrd. Euro ergeben. Muss der Staat Zinsen z. B. in Höhe von 2,5 % für die Kredite zahlen, müsste die Rendite rund 16 % betragen. Bei geringeren Renditen würde die zukünftige Generation durch die Änderung des Tilgungsplans belastet.

## Zusammenfassung und Ausblick

Im Fall von Naturkatastrophen darf die Bundesregierung von der im Grundgesetz geregelten sogenannten Schuldenbremse abweichen. Allerdings ist nach Art. 115 Abs. 2 Satz 7 und 8 ein Tilgungsplan mit einer angemessenen Tilgungszeit aufzustellen. Nach geltendem Recht wird der Bund im Jahr 2023 mit der Tilgung der Kredite, die im Zusammenhang mit der COVID-19-Pandemie aufgenommenen wurden, beginnen. Abgeschlossen sein soll die Tilgung im Jahr 2042. Die neue Bundesregierung möchte laut Koalitionsvertrag aber erhebliche Änderungen bei diesen Tilgungsplänen vornehmen. Diese Vorgehensweise rechtfertigt sie mit einer Orientierung an dem Programm „Next Generation EU", obwohl für diese Orientierung keine sachliche Notwendigkeit besteht. Wie eine solche neue Tilgungsregelung im Detail aussehen wird, ist bisher unklar. Es ist aber davon auszugehen, dass sich die Koalition wie eine stimmenmaximierende Politikerin verhalten und deshalb die Tilgung strecken wird. Von den hier betrachteten drei Tilgungsszenarien erhöht die Variante für die Bundesregierung den aktuellen Einnahmenspielraum am stärksten, bei der die Tilgung auf die Jahre 2028 bis 2058 verschoben wird, so wie es auch bei der EU vorgesehen ist. Dieser Spielraum beträgt in der aktuellen Legislaturperiode 6,3 Mrd. Euro; in der 21. Legislaturperiode wären es 59,4 Mrd. Euro.

Eine Streckung der Tilgung führt bei einer vorgegebenen Tilgungssumme zu einer Minderung der jährlichen Tilgungsleistung in den ursprünglichen Tilgungsperioden, was wiederum den Ausgabespielraum in diesen Perioden erhöht. Dafür werden in späteren Perioden Tilgungsleistungen zu erbringen seien, in denen es nach dem Status quo nicht der Fall ist. Eine Verlängerung des Tilgungszeitraums führt dazu, dass Zinsänderungsrisiken über einen längeren Zeitraum wirken können. Derzeit verschuldet sich die Regierung vor allem kurzfristig. Beide Aspekte, verlängerter Tilgungszeitraum und kurzfristige Verschuldung, stellen in einem Umfeld steigender Renditen, wie es sich momentan abzeichnet, ein großes finanzielles Risiko dar.

Eine Verlängerung des Tilgungszeitraums berührt nicht nur das Zinsänderungsrisiko, sondern führt auch zu einer Verlagerung von Zahlungslasten in die Zukunft. Wird der Tilgungszeitraum der EU übernommen, würden im Zeitraum 2043 bis 2058 noch 184 Mrd. Euro der im Zusammenhang mit der Coronakrise aufgenommenen Kredite getilgt werden müssen. Würde die Regierung die sich durch die Tilgungsstreckung ergebenden finanziellen Spielräume vollumfänglich für öffentliche Investitionen verwenden („Goldene Regel"), so ergäbe sich keine Lastverschiebung auf Kosten der späteren Generation. Allerdings ist anzuzweifeln, dass die Regierung die sich ergebenden Mehreinnahmen tatsächlich vollumfänglich für Investitionen verwenden wird, sodass es zu einer Lastverschiebung in die Zukunft käme.

Mit diesem Beitrag sollte gezeigt werden, dass die in Art 115 Abs. 2 GG vorgesehene Ausnahmeregel von der Schuldenbremse sich auch bei der Schuldentilgung als

3 Es wird unterstellt, dass die sich bei einem Zinssatz von -0,65 % in den Jahren 2023 bis 2042 ergebenden Mehreinnahmen (38,5 Mrd. Euro – 27,4 Mrd. Euro) aus der Tilgungsstreckung der Gegenwartsgeneration zufließt. Die Mehrausgaben bei einem Zinssatz von 2,5 % hingegen werden der zukünftigen Generation aufgebürdet.

problematisch erweisen kann, wenn wählerstimmenmaximierende Politiker:innen unterstellt werden. Regulierend eingreifen kann das z. B. von der Opposition angerufene Bundesverfassungsgericht. Da dieses aber vor allem die Folgerichtigkeit prüft, um den weiten Ermessenspielraum der Gesetzgebung nicht einzuschränken, ist der Effekt unklar. Alternativ könnte gesetzlich geregelt werden, dass für die Aufstellung des Tilgungsplans die absolute Mehrheit nicht ausreicht und stattdessen ein höheres Quorum (z. B. eine 2/3-Mehrheit) erforderlich ist (Sachverständigenrat, 2007, 101). Ein weiterer einfacher Ansatz könnte darin bestehen, einen maximalen Tilgungszeitraum (z. B. 20 oder 25 Jahre) festzulegen, um die Lastverschiebung auf folgende Generationen zu vermeiden bzw. zu erschweren. In diesem Fall könnte keine Regierung sich zusätzliche Einnahmenspielräume durch Streckung der Tilgung verschaffen. Für die Ausgestaltung der jährlichen Raten sind dann verschiedene Ansätze denkbar (Feld et al., 2021, 338-340).
